# Investigating the Relationship Between Highly Collaborative Local Health Jurisdictions and County-Level Perinatal Health Outcomes

**DOI:** 10.1007/s10995-026-04321-9

**Published:** 2026-09-21

**Authors:** Amelia L. Mitchell, Taryn A. G. Quinlan, Glen P. Mays

**Affiliations:** 1 Department of Health Systems, Management & Policy, Colorado School of Public Health, University of Colorado Anschutz Medical Campus, Aurora, CO USA; 2 RWJF Systems for Action Research Program, University of Colorado, Aurora, CO USA

**Keywords:** Perinatal health, Collaboration, Health equity

## Abstract

**Objectives:**

Clarifying how cross-sector collaboration relates to perinatal health outcomes is essential in establishing tools to improve maternal and infant health. This study aims to establish if residence in a highly collaborative local health jurisdiction relates to one’s probability of experiencing poor perinatal health outcomes.

**Methods:**

We measured collaboration between local health departments and social service organizations using data from the 2018 National Longitudinal Survey of Public Health Systems (NALSYS). We then used Latent Class Analysis to identify high and low collaborating counties, followed by multiple logistic regressions to assess the probability of six selected perinatal health outcomes among those residing in high and low collaborating counties at the time of labor and delivery. Health outcome data was selected from the 2018 National Vital Statistics System (NVSS) data.

**Results:**

Using a national sample of local health departments across the United States, we found significant racial differences in the association between residence in a high collaborating county and the probability of poor perinatal health outcomes. White women experienced a lower probability of poor perinatal health outcomes if they resided in a high versus a low collaborating county, whereas the inverse was true for Black women. When stratified by payer, privately insured individuals had a lower risk of gestational diabetes if they resided in a high collaborating county, whereas those covered by Medicaid saw little to no effect.

**Conclusions for Practice:**

These results suggest that local health jurisdictions should give greater attention to the needs of historically marginalized populations when planning and implementing collaborative activities to address community health needs.

## Significance

Cross-sector collaboration and partnerships have been found to improve population health and have been touted as a potential strategy to improve perinatal health outcomes. However, no prior study has explored this topic quantitatively. Despite hopes that collaboration would help mitigate social determinants that result in poor health outcomes, this study finds that the benefits of collaboration may accrue disproportionately to White and privately insured populations. These results suggest a need for collaborative activities that focus more deliberately on addressing racial and socioeconomic inequities in perinatal health.

## Introduction

Despite spending the most on health care, the United States continues to have the worst maternal and infant mortality rates among high-income countries (Gunja et al., 2023). Critically, a substantial proportion of these deaths are preventable: national reviews have found that up to 84% of pregnancy-related deaths could be prevented with improvements to care quality, access, and coordination (Trost et al., 2022). The COVID-19 pandemic further increased maternal mortality rates and widened existing racial disparities in perinatal outcomes (Simpson, 2023). These disparities are particularly stark for Black women, who die from pregnancy-related causes at approximately three times the rate of White women — a disparity that persists across income and education levels and is largely attributable to preventable causes (Hoyret, 2024). Additionally, the reversal of Roe v. Wade has increased obstacles to safe abortion care, which may disproportionately impact people of color and worsen disparities in perinatal health (Dehlendorf et al., 2013; Farin et al., 2021). Addressing these crises requires a multipronged approach that strengthens economic and social supports before, during, and after pregnancy, and improves access to, coverage, and quality of care (*White House Blueprint addressing the Maternal Health Crisis*, 2022). Collaborations between local health and social service organizations may represent one such approach.

There is limited but consistent evidence that systems collaboration can improve population health outcomes. Recent research finds that public health jurisdictions that expand their multisector collaboration networks see a decrease in preventable deaths (Mays et al., 2016). Collaboration is also associated with reduced emergency department visits and inpatient hospitalization (Ostovari & Yu, 2019). Qualitative evidence suggests that collaborative partnerships are key to success in local public health jurisdictions with exceptionally good perinatal health outcomes (Klaiman et al., 2016). In practice, programs that promote collaboration between social services and local public health entities see improved perinatal health outcomes. Notably MAMA’s Neighborhood, a Los Angeles based initiative, has seen success at increasing hospital-based deliveries and postpartum care through local public health and social service collaborations (Saleeby et al., 2021). At the national level, Healthy Start — the largest federally funded initiative explicitly focused on reducing infant mortality and racial and ethnic disparities in perinatal health outcomes — has long promoted collaboration between local health departments, community organizations, and social service agencies as a core program strategy (Health Resources & Services Administration, 2025). While Healthy Start and similar national MCH programs provide important anecdotal and programmatic evidence of the value of collaboration, rigorous quantitative studies examining the relationship between systems collaboration and county-level perinatal outcomes using standardized collaboration metrics remain limited. The present study seeks to address this gap.

Collaboration between local organizations and the local health department has the potential to improve perinatal health outcomes by pooling resources, decentralizing care, and addressing upstream social determinants of health (e.g., education, economic stability, built environment, and healthcare access) (Crear-Perry et al., 2021). This is accomplished through strategic partnerships that allow organizations to provide services that they may not have the resources, time, or expertise to supply independently (Mays & Scutchfield, 2010). Prior studies have emphasized the specific importance of community partnerships in helping to improve maternal and child health (MCH) outcomes (Klaiman et al., 2016; Kroelinger et al., 2012; Morgan et al., 2020). However, no prior study has investigated how collaboration at the county level in the delivery of essential public health services relates to perinatal health.

Our research group has previously investigated collaboration characteristics among counties with and without MCH delivery at the local health department (LHD) level (Quinlan et al., 2023). Through this work, we found that LHDs that provide MCH services exhibit greater cross-sectoral collaboration between community organizations, LHDs, and social service sectors, than those that do not, suggesting improved system capacity (Quinlan et al., 2023). We also found that rural and highly vulnerable communities have the lowest level of collaboration (Quinlan et al., 2023). This prior study used an identical sample, definitions of high and low collaboration, and latent class analysis approach as employed in the present study, allowing for direct comparability across findings. This research identified which counties might require further investment in systems collaboration. However, as collaborative partnerships require time and resources, understanding how they relate to perinatal health outcomes is critical as we look towards cross-sector collaboration as a potential strategy to address current perinatal health crises.

We build upon this prior research by investigating how cross-sectoral collaboration relates to perinatal health outcomes in counties with direct MCH service provision by the LHD. This study seeks to understand the relationship between local public health collaboration in the delivery of essential public health services and key maternal and infant health outcomes, and to examine whether these outcomes vary by race, ethnicity, and payer type.

## Methods

### Data and Study Population

We conducted a cross-sectional study to assess the probability of six selected perinatal health outcomes among individuals residing in US counties with low and high levels of collaboration in the delivery of essential public health activities 2018. We included individuals with available birth, pregnancy, and covariate data in 2018 and who resided in high or low collaboration counties, as defined below.

We measured cross sector collaboration using 2018 National Longitudinal Survey of Public Health Systems (NALSYS) data. The NALSYS has followed a nationally representative cohort of jurisdictions served by local public health agencies in the United States since 1998 (*n* = 776) (Mays, 2020). The 2018 NALSYS received a 71% response rate. The NALSYS measures collaboration by asking LHDs to report which community organization types are involved in the provision of a selected set of 20 essential public health activities. Potential collaboration sectors include local and state health agencies, medical providers, faith-based organizations, and non-profits, among others (Appendix Table 4). The 20 essential public health activities observed were selected to encompass the three core functions of public health presented by the Institute of Medicine’s 1988 consensus report on the US public health system (Mays, 2020; Walker, 1989). The NALSYS also measures whether the collaborators observed provide any of 18 possible social services as a part of the provision of that activity (Appendix Table 5). We created an overall metric of collaboration by measuring the percent of essential public health activities delivered in collaboration between the LHD and community organizations that also deliver social services.

Our sample was restricted to counties with LHDs that reported providing MCH services. This designation was determined using 2016 National Association of County and City Health Officials (NACCHO) data wherein data on specific LHD service provision has been collected over time. Direct MCH service provision included family planning, prenatal care, obstetrical care, WIC, MCH home visits, Early and Periodic Screening, Diagnostic, and Treatment, and well child clinic. Restricting our sample to LHDs that provide MCH services directly builds upon our previous work, wherein we identified higher levels of collaboration among counties with direct MCH service provision, (Quinlan et al., 2023). by evaluating if higher levels of collaboration in these counties bear a relationship with perinatal health outcomes. NALSYS and NACCHO data were linked by identifiers of the responding local health department. Additionally, we excluded multi-county jurisdictions, where one LHD serves multiple counties, and counties with multiple LHDs surveyed by the NALSYS. LHDs are a diverse sample population with a wide variety of organizational structures and duties. Restricting our sample to those that serve a single county and are directly involved in MCH service provision reduces the heterogeneity in our county and LHD sample. Excluding counties with multiple LHDs represented by the NALSYS prevented us from double counting one county and thereby biasing our results.

A total of 527 counties had both NACCHO and NALSYS data for the corresponding data years and an LHD that reported providing maternal and/or child health services.

### Comparison Groups

We identified counties as low versus high collaborators using latent class analysis (LCA). LCA reduces heterogeneity between diverse groups, such as LHDs, by using observed variables to identify qualitatively distinct latent subgroups within a defined sample (Weller et al., 2020). Our LCA identified two mutually exclusive groups within the study population, referred to in the present study as “high” and “low” collaborating counties. The LCA used in this study was originally performed on an identical sample used to define patterns of engagement among LHDs that provide MCH services (Quinlan et al., 2023).

We then used county residence status at the individual level, taken from 2018 National Vital Statistics System (NVSS) data, to divide our sample into individuals that resided in “low” and “high” collaborating counties at the time of delivery. Individuals from the NVSS data were matched to their residential counties using federal information processing standards (FIPS) codes.

### Outcomes

We measured perinatal health outcomes using 2018 NVSS data. The NVSS is an official governmental dataset, collected and disseminated by the National Center for Health Statistics, that aggregates statistics on vital life events in the United States, including pregnancy and birth outcomes (Statistics, 2016). Selected NVSS outcomes included in the present study were (1) gestational diabetes, (2) gestational hypertension, (3) maternal intensive care unit (ICU) admission, (4) low birth weight, (5) preterm birth, and (6) neonatal intensive care unit (NICU) admission. Infants were categorized as having a low birth weight if they were born weighing less than 5.5 pounds or 2500 g, and preterm if they were born before 37 weeks of gestation, consistent with World Health Organization’s definitions (Low Birth Weight, 2024; *Preterm birth*, 2023).

### Statistical Analyses

We conducted a logistic regression for each selected perinatal health outcome. The primary predictor was residence in a county with a high level of collaboration in the delivery of essential public health activities. Individuals with pregestational diabetes or pregestational hypertension were excluded from analyses predicting the probability of gestational diabetes and gestational hypertension, respectively.

We conducted both unadjusted and adjusted logistic regression models, clustering standard errors at the state and county levels to account for unobserved geographic differences. Our adjusted model controlled for county and individual level demographics (Table 1). Individual demographics were selected from NVSS data. County level demographics were selected from the 2018 Area Health Resource File (AHRF) and linked using FIPS codes. We also adjusted for the maternal vulnerability index (MVI) at the county level, which is a weighted index of geographic vulnerability to poor perinatal health outcomes (Appendix Table 6) (Ventures, 2021). We also adjusted for the interaction between maternal race/ethnicity and collaboration status. Marginal effects of the interaction between race and collaboration status were calculated using Buis’ method of extracting marginal effects for interaction terms in a logistic regression model (Buis, 2010).

**Table 1:** Descriptives statistics in high and low collaborating counties

	Low collaborating county residence	High collaborating county residence
	*N* = 347,824	*N* = 718,699
*Individual Demographics*		
*Mother’s Race and Ethnicity*		
Non-Hispanic White (only)	196,343 (56.4%)	405,672 (56.4%)
Non-Hispanic Black (only)	41,915 (12.1%)	91,876 (12.8%)
Non-Hispanic American Indian Alaska Native (only)	2,743 (0.8%)	3,123 (0.4%)
Non-Hispanic Asian (only)	20,940 (6.0%)	44,778 (6.2%)
Non-Hispanic Native Hawaiian or Pacific Islander (only)	710 (0.2%)	1,829 (0.3%)
Non-Hispanic more than one race	6,903 (2.0%)	15,826 (2.2%)
Hispanic	78,270 (22.5%)	155,595 (21.6%)
*Mother’s Nativity*		
Born in the US	269,093 (77.4%)	540,520 (75.2%)
Born outside the US	78,731 (22.6%)	178,179 (24.8%)
First birth	130,440 (37.5%)	279,210 (38.8%)
*Mother’s Educational Attainment*		
Less than high school	34,822 (10.0%)	71,782 (10.0%)
High School degree or GED	81,563 (23.4%)	158,589 (22.1%)
Some college	67,223 (19.3%)	136,248 (19.0%)
Associate degree	30,469 (8.8%)	64,396 (9.0%)
Bachelor’s Degree or more	133,747 (38.5%)	287,684 (40.0%)
*Father’s Educational Attainment*		
Less than high school	40,661 (11.7%)	82,099 (11.4%)
High School degree or GED	102,662 (29.5%)	197,721 (27.5%)
Some college	65,736 (18.9%)	132,755 (18.5%)
Associate degree	25,752 (7.4%)	55,060 (7.7%)
Bachelor’s Degree or more	113,013 (32.5%)	251,064 (34.9%)
Mother’s Age (Reported)	29 (6)	30 (6)
Father’s Age (Combined computed and reported age of father)	32 (7)	32 (7)
*Payment Type*		
Medicaid	123,902 (35.6%)	252,265 (35.1%)
Private Insurance	192,140 (55.2%)	413,012 (57.5%)
Self-Pay	15,774 (4.5%)	28,752 (4.0%)
Other	16,008 (4.6%)	24,670 (3.4%)
*Mother’s marital status*		
Married	241,879 (69.5%)	496,696 (69.1%)
Unmarried	105,945 (30.5%)	222,003 (30.9%)
*Risk Factors*		
Pre-existing diabetes	3,214 (0.9%)	6,807 (0.9%)
Pre-existing hypertension	7,435 (2.1%)	13,665 (1.9%)
*County-level Demographics*		
*Maternal Vulnerability Index*		
Very low	106,806 (30.7%)	190,688 (26.5%)
Low	40,224 (11.6%)	149,033 (20.7%)
Moderate	76,198 (21.9%)	206,941 (28.8%)
High	108,278 (31.1%)	149,577 (20.8%)
Very High	16,318 (4.7%)	22,460 (3.1%)
Rural	32,302 (9.3%)	21,606 (3.0%)
Obstetricians (per 1000)	13 (7)	13 (6)
Unemployment Rate (%)	4 (1)	4 (1)
Population Density (per 1000 per square mile)	1 (1)	1 (2)

We then conducted stratified logistic regressions by payer (Medicaid and Private insurance, excluding uninsured and other) and maternal race (non-Hispanic Black Individuals and non-Hispanic White Individuals, excluding other race and ethnicity groups) and used seemingly unrelated estimation techniques to compare coefficient estimates across stratified models. In all analyses, counties were weighted to account for stratified sampling design in the NALSYS data.

This research was conducted in accordance with prevailing ethical principles. No institutional review board was required as this research relied on secondary data use and all identifiable participant information was removed prior to data receipt. This study was reviewed by the Colorado Multiple Institutional Review Board (COMIRB) and determined to be not human subject’s research.

## Results

A total of 1,066,523 observations with available birth, covariate, and county level data during the 2018 study year period were included in this analysis. Descriptive statistics for individuals in low (*n* = 347,824) and high (*n* = 718,699) collaborating counties are presented in Table 1. Demographic statistics were overall similar in high and low collaborating counties (Table 1). However, those in low collaborating counties were more likely to reside rurally, at 9.3% compared to 3.0% with rural residence in high collaborating counties (Table 1). Those in low collaborating counties were also more likely to reside in a county with a high or very high maternal vulnerability index (35.8%) compared to those in high collaborating counties (23.9%) (Table 1).

### Pregnancy Outcomes

#### Gestational Diabetes

Neither our adjusted or unadjusted model results found a significant association between high collaborating county residence and gestational diabetes (Table 2). Using Buis’ method of calculating marginal effects for interaction terms^6^, we found that the marginal effect of high collaborating county residence on gestational diabetes probability was 0.46%-points lower for white individuals (SE = 0.28, *p* < 0.1) and 0.70%-points lower for Black individuals (SE = 0.36, *p* < 0.05) residing in a high versus a low collaborating county. Those with a preexisting diabetes diagnosis were excluded from this analysis (Table 3).

**Table 2:** Unadjusted and adjusted^1^ marginal effects of residing in a high collaborating county on perinatal health outcomes

	Unadjusted model	Adjusted model
*Maternal Outcomes*		
Gestational Diabetes	−0.00341 (0.00294)	−0.00424 (0.00257)
Gestational Hypertension	−0.00117 (0.00427)	0.00131 (0.00319)
Maternal ICU Admission	−0.0000117 (0.000144)	0.0000352 (0.000149)
*Infant Outcomes*		
Low weight birth	−0.00220 (0.00315)	−0.000859 (0.00121)
Preterm birth	−0.00293 (0.00378)	−0.00197 (0.00197)
NICU Admission	−0.00193 (0.00440)	−0.00458 (0.00333)

1. Our adjusted model uses logistic regression to adjust for all covariates listed in Table 1 and the interaction between high collaboration and race and ethnicity

#### Gestational Hypertension

There was no significant marginal effect of residence in a high collaborating county on the probability of experiencing gestational hypertension in either the adjusted or unadjusted logistic regression models (*p* > 0.1). Those with preexisting hypertension were excluded from this analysis (Table 2). There was no significant marginal effect of the interaction between race and high collaborating county residence and the probability of gestational hypertension (Table 3).

**Table 3:** Marginal effect of collaboration for white and black pregnant individuals using Buis' method of extracting marginal effects for interaction terms^17^

	White individuals	Black individuals
*Maternal Outcomes*		
Gestational Diabetes	−0.0046* (0.0028)	−0.0070** (0.0036)
Gestational Hypertension	0.0002 (0.0042)	0.0046 (0.0081)
Maternal ICU Admission	0.0002(0.0002)	−0.0002 (0.0003)
*Infant Outcomes*		
Low weight birth	−0.0041*** (0.0016)	0.0087** (0.0036)
Preterm birth	−0.0066** (0.0026)	0.0093** (0.0047)
NICU Admission	−0.0024 (0.0040)	0.0047 (0.0085)

#### Maternal ICU Admission

There was no significant marginal effect of residence in a high collaborating county on the probability of maternal ICU admission in either the adjusted or unadjusted logistic regression models (*p* > 0.1) (Table 2). There was no significant marginal effect of the interaction between race and high collaborating county residence and the probability of maternal ICU admission (Table 3).

### Infant Outcomes

#### Low-Weight Birth

We found no significant marginal effect of residence in a high collaborating county on the probability of low weight birth. However, we did see a non-significant decrease in both adjusted and unadjusted models (Table 2). There was a significant marginal effect of the interaction between race and collaboration, such that white individuals in a high collaborating county saw a 0.41%-point decrease (SE = 0.16, *p* < 0.01) and Black individuals saw a 0.87%-point increase (SE = 0.36, *p* < 0.05) in the probability of low weight birth if they resided in a high versus a low collaborating county (Table 3).

#### Preterm Birth

Our preterm birth results were very similar to those in our low weight birth analysis, showing a non-significant negative effect of high collaborating county residence (Table 2). We found a significant marginal effect of the interaction between race and collaboration status such that white individuals in a high collaborating county saw a 0.66%-point decrease (SE = 0.26, *p* < 0.05) and Black individuals saw a 0.93%-point increase (SE = 0.47, *p* < 0.05) in the probability of preterm birth (Table 3).

#### NICU Admission

There was no significant association between residence in a high collaborating county and the probability of NICU admission (*p* > 0.1) (Table 2). Additionally, we found no significant marginal effect of the interaction between race and collaboration status (Table 3).

### Race and Payer Stratification

After completing our adjusted and unadjusted logistic regression models, we stratified observations by reported maternal race, ethnicity and payer and repeated our adjusted logistic regression for each of the selected perinatal health outcomes. Seemingly unrelated estimation techniques were used to compare coefficients across models.

**Fig. 1:**
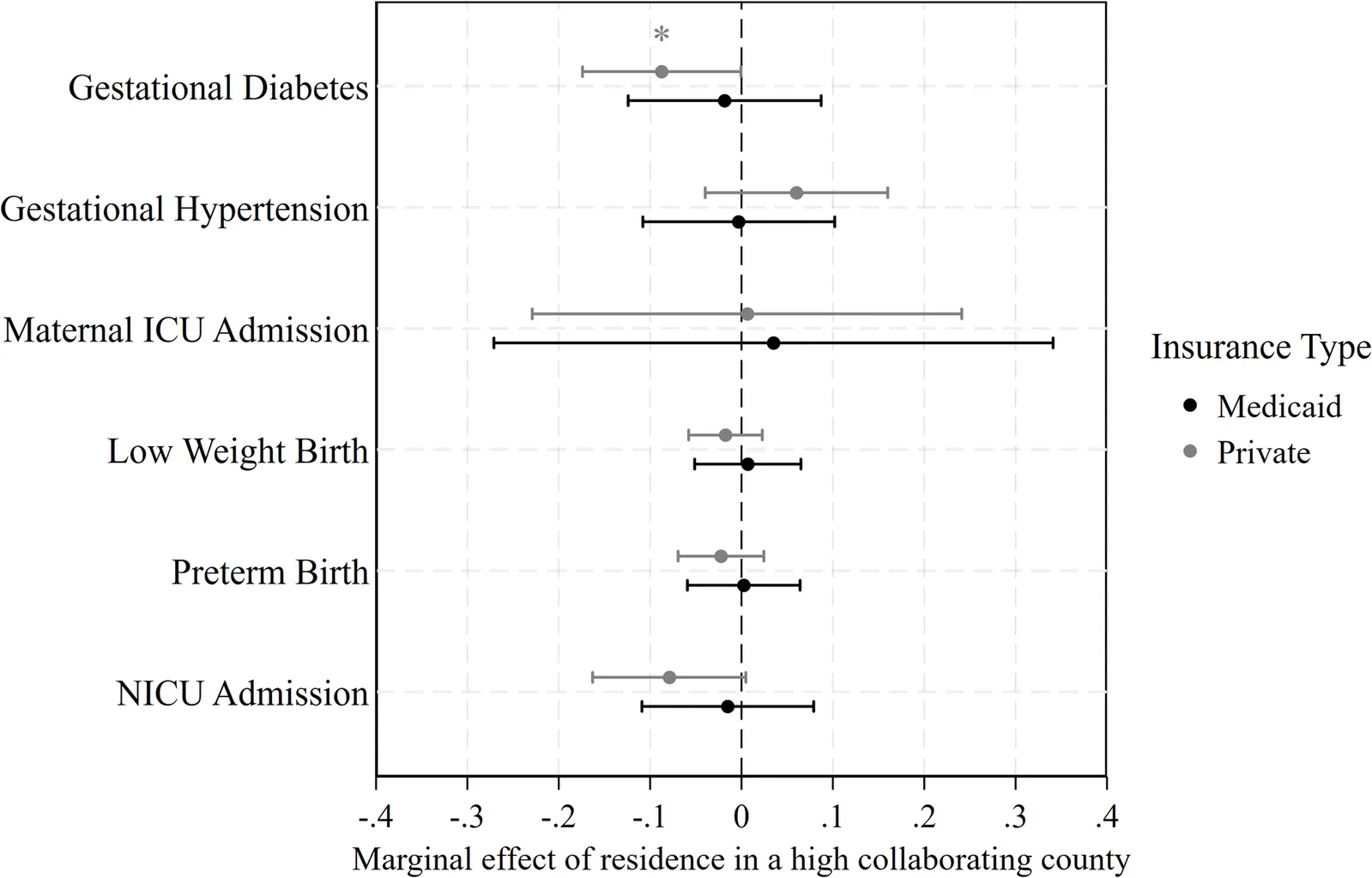
Marginal effect of residence in a high collaborating county as it relates to the probability of selected perinatal health outcomes, stratified by payer type (private insurance or Medicaid). Clustered standard errors were used in this analysis. Error bars represent 95% confidence interval. ***p <0.01, **p <0.05, *p <0.1

Privately insured individuals residing in a high collaborating county saw an 8.75%-point decrease in the probability of gestational diabetes (SE = 4.44, *p* < 0.1) compared to those residing in a low collaborating county. Individuals whose births were covered by Medicaid, however, saw no significant marginal effect of residence in a high collaborating county on any of the perinatal health outcomes observed (Fig. 1).

**Fig. 2:**
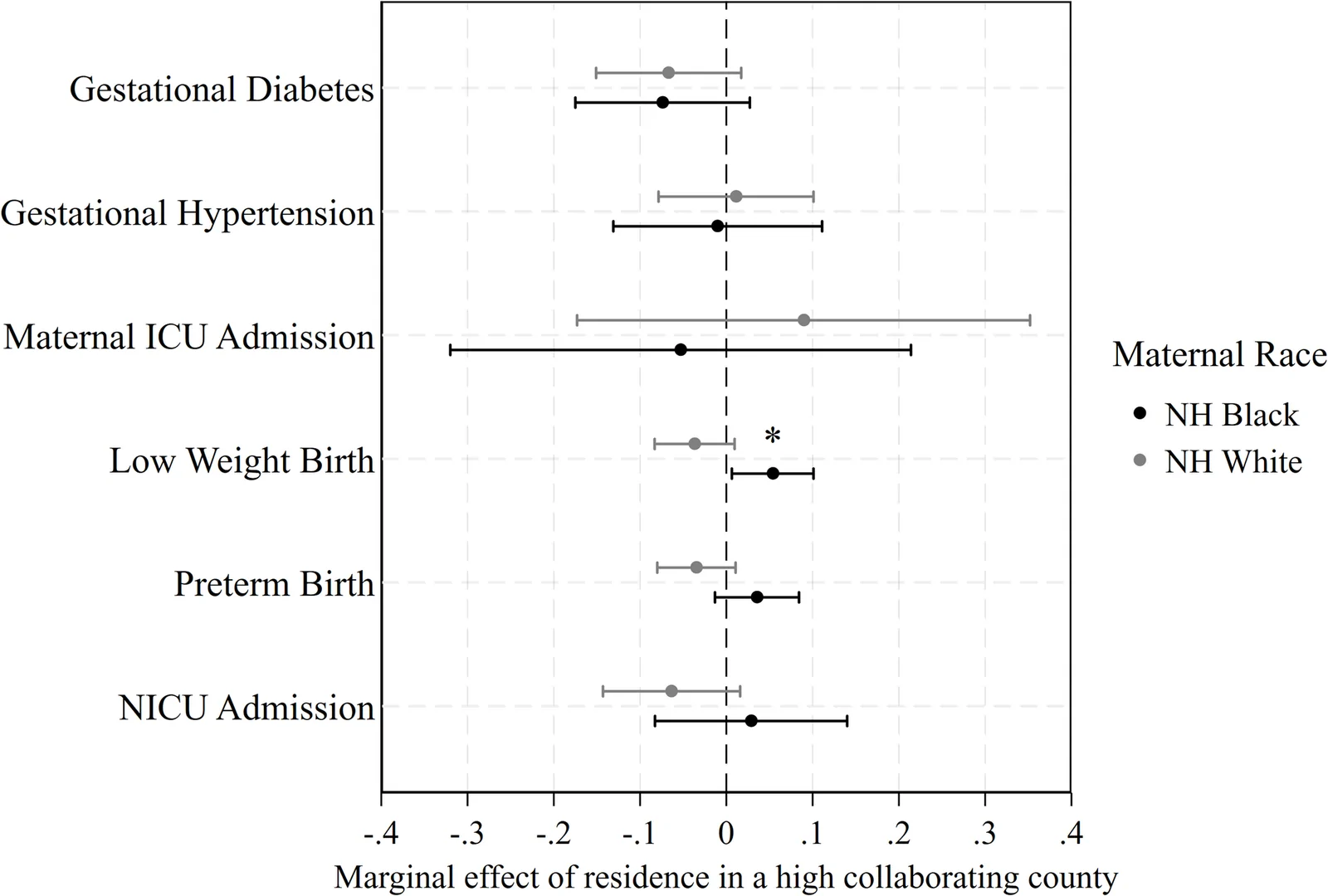
Marginal effect of residence in a high collaborating county as it relates to the probability of selected perinatal health outcomes, stratified by race (non-Hispanic Black or non-Hispanic White). Clustered standard errors were used in this analysis. Error bars represent 95% confidence interval. ***p <0.01, **p <0.05, *p <0.1

In our fully stratified model, there was no significant association between high collaborating county residence and perinatal health outcomes among those with reported non-Hispanic White maternal race. However, non-Hispanic Black individuals residing in a high collaborating county experienced a 5.39%-point increase in the probability of low weight birth, compared to those residing in a low collaborating county. Among non-Hispanic White individuals, estimates suggest a directional but non-significant decrease in the probability of poor infant health outcomes if they resided in a high collaborating versus a low collaborating county. In contrast, for non-Hispanic Black individuals, estimates indicated a directional but non-significant increase in the probability of poor infant health outcomes associated with high-collaborating county residence. Both non-Hispanic Black and non-Hispanic white individuals appeared to have a decreased probability of gestational diabetes if they resided in a high-collaborating county, but this effect also did not reach significance (Fig. 2).

## Discussion

The association between collaboration in the delivery of essential public health activities and perinatal health outcomes varies significantly by race and payer. Marginal effects of the interaction between race and collaboration show that non-Hispanic Black individuals have an increased probability of low birthweight and preterm birth if they reside in high versus low collaborating counties, while non-Hispanic white individuals in high collaborating counties show a decreased probability of both (Table 3). These disparities persist in race-stratified models (Fig. 2). When stratified by payer, privately insured individuals see a lower probability of gestational diabetes in high collaborating counties. However, those covered by Medicaid see no effect. Together, these results suggest that the potential perinatal health benefits of collaboration are underrealized and unequally distributed.

Prior research has found that collaboration and multisectoral partnerships have the potential to improve population and perinatal health by pooling resources and addressing upstream social determinants of health (Klaiman et al., 2016; Mays et al., 2016; Ostovari & Yu, 2019). These findings help to explain the inverse association between high collaboration and gestational diabetes among privately insured populations found in the present study. However, they do not explain the lack of benefit among Medicaid enrollees or the greater risk of poor perinatal health outcomes among Black individuals in high collaborating counties.

Economic and racial disparities in perinatal health outcomes are largely attributed to linked social determinants, including education, income, environmental exposure, and psychosocial stress (Burris & Hacker, 2017). Systems collaboration may address some of these determinants, but may be insufficient to counteract the disproportionately poor perinatal and infant health outcomes experienced by Black and low-income individuals. The United States’ history of segregation and discrimination has resulted in widespread systemic racism, which, through various channels, has adversely impacted lifelong stress exposure and perinatal health outcomes among Black communities (Burris & Hacker, 2017). Similarly, lower socioeconomic status, like that experienced among many Medicaid enrollees, can cause substantial stress and force individuals to make tradeoffs between essential needs and health care over the life course (Girardi et al., 2023).

Collaboration between the local health department and social service organizations may address proximal barriers to perinatal health, such as access to prenatal care, food and housing security. LHDs can seek to improve collaborations through partnership building and community outreach. However, collaboration may not be sufficient to address distal factors such as lifelong psychosocial stress and discrimination. Further examination of potential mediating outcomes, such as prenatal care attendance or food and housing availability, may help to better understand whether the lack of benefit of collaboration observed among Black and Medicaid populations is due to distal upstream determinants or if existing collaborations in service delivery exclude these populations directly.

The increased risk of low birthweight observed among Black individuals in highly collaborative counties warrants further investigation. This increase, and the overall lack of benefit for Black mothers, could be explained by reverse causality- counties with existing poor Black perinatal health outcomes may be more likely to increase their collaboration and community partnerships. More likely, however, is that racial segregation in service availability within the counties observed allows collaborations to improve outcomes for white populations while disadvantaging Black populations. Recent research on infant mortality finds that higher levels of structural racism and inequality in urban counties leads to decreased mortality for white populations and increased mortality for Black populations (Vilda et al., 2021). Although we adjusted for rurality and clustered at the state and county level, we did not directly measure structural racism or levels of geographic segregation, which may differ between high and low collaborating counties. Further research should examine how collaboration interacts with structural racism and their joint association with perinatal health outcomes.

The strengths of this study lie in our use of the nationally representative NALSYS data to assess local public health collaboration. The use of an LCA to determine collaboration groups also aids in our ability to control for latent confounders. Further, the consistency in results observed across race and payer categories supports the likelihood that the differential relationship between collaboration and perinatal health outcomes observed is a real effect associated with being a member of a less versus more marginalized group. The causal interpretation of this study, however, is limited by its cross-sectional design. While prior longitudinal and qualitative research has uncovered positive effects of collaboration on population health outcomes, (Klaiman et al., 2016; Mays et al., 2016; Ostovari & Yu, 2019) our cross-sectional study does not address causality. Further, we caution against generalizing these results to counties without direct MCH service provision by the LHD, as our sample was restricted to a subset of LHDs with direct MCH service provision. Additionally, the data in this study were from before the COVID-19 pandemic. Perinatal health outcomes worsened and racial and ethnic disparities widened over the course of the pandemic in the United States (Simpson, 2023). Assessing the relationship between collaboration and perinatal outcomes prior to the pandemic has allowed us to assess this relationship under non-emergency conditions. However, we suggest caution in generalizing them to a post-pandemic context. We were also unable to measure the quality of care at the county level, so this potential bias remains and may be a key focus of further research.

## Conclusion

The relationship between perinatal health outcomes and multi-sector collaboration in the delivery of essential public health activities is inconsistent across race and payer groups. These results suggest that local public health collaboration efforts should give greater attention to addressing inequities in perinatal health outcomes and the specific needs of historically marginalized population groups. Further work is needed to identify how best to utilize collaboration to better benefit non-Hispanic Black individuals and Medicaid enrollees and improve perinatal health outcomes.

## Appendix

Tables 4, 5 and 6.

**Table 4:** Measured collaboration sectors included in the 2018 NALSYS

Sector type	Sector name
Local or State Health Agency	Local Health Department
	State Health Agency
Other Governmental or Local Agencies	Other State Agencies
	Local Agencies
	Federal Agencies
Medical	Physician Organizations
	Hospitals
	Health Insurers
	Community Health Centers
Education	School (Kindergarten-12th grade)
	Colleges/universities
Other	Faith-Based Organizations
	Employers/Business groups
	Tribal Organizations
	Other Nonprofit Organizations
	Other

**Table 5:** Measured social service sectors included in the 2018 NALSYS

Service category	Social service type
Basic Needs	Cash assistance
	Economic Development
	Employment assistance or job training
	Food and nutrition assistance
	Housing, shelter or utility assistance
	Transportation
Criminal Justice	Corrections and criminal justice
	Law enforcement
	Legal assistance
Culture and Recreation	Arts, library, or cultural
	Parks and recreation
Land Use and Environment	Agricultural or cooperative extension
	Environmental protection
	Land use, zoning or community development
Mental Health and Addiction Services	Mental health
	Substance abuse
Social support Services	Child and family support
	Support services for disabled populations
	Support services for older adults
	Support services for veterans

**Table 6:** Maternal vulnerability index architecture^a^

Theme	Subtheme	Indicator
1. Reproductive Healthcare	Family Planning Needs	Female contraceptive clients served at publicly funded clinics per 100k women of reproductive age, aged 13–44
	Access to Abortions	Minimum distance to nearest abortion clinic
		Count of supportive abortion policies
	Public Reproductive Health Funding	Public expenditures for family planning client services
	Reproductive Health System Capacity	Ratio of ob-gyn providers
		Ratio of Nurse Midwives
		Ratio of newborn bassinets
2. Physical Health	Hypertension	Percent with high blood pressure
	Diabetes	Percent with diabetes
	Obesity	Percent that is obese
	Prevalence of STIs	Prevalence of gonorrhea
		Prevalence of primary or secondary syphilis
		Prevalence of chlamydia
		Prevalence of hepatitis B
	HIV Prevalence	Prevalence of HIV
	Self-Rated Health	Percent reporting poor or fair health
3. Mental Health and Substance Abuse	Stress	Percent reporting frequent mental distress
		Percent with a long commute (> 30 min)
	Mental Health Status	Percent reporting any mental illness
		Percent ever diagnosed with depression
	Accessibility	Mental health providers
	Substance Abuse	Percent that smoke
		Age adjusted overdose death rate
4. General Healthcare	Affordability	Postpartum extension status
		Income eligibility limit for pregnant individuals
		Medicaid expansion status
		Percent who are uninsured
	Accessibility	Distance to nearest hospital
	Care seeking Behavior	Percent that reported a routine physical checkup
		Percent that reported having a primary care provider
	Quality	Prevention Quality Indicator (PQI)
5. Socioeconomic Determinants	Educational Attainment	Percent with a bachelor’s degree
		Percent with no high school degree
	Minority Status	Percent that speaks English less than well
	Poverty	Percent that live under poverty
	Food Insecurity	Percent suffering from food insecurity
	Social Capital	Percent that are single female headed
		Social capital index
6. Physical Environment	Housing	Percent with severe housing problems
	Violence	Violent crime rate
	Transportation	Percent with no access to a vehicle
	Air pollution	Transit Connectivity Index (TCI)
		Concentration of PM2.5

## References

[CR1] Buis, M. L. (2010). Stata tip 87: Interpretation of interactions in nonlinear models. *The stata journal*, *10*(2), 305–308.

[CR2] Burris, H. H., & Hacker, M. R. (2017). Birth outcome racial disparities: A result of intersecting social and environmental factors. *Seminars In Perinatology*, *41*(6), 360–366. 10.1053/j.semperi.2017.07.002 PMC5657505 28818300

[CR3] Crear-Perry, J., Correa-de-Araujo, R., Lewis Johnson, T., McLemore, M. R., Neilson, E., & Wallace, M. (2021). Social and Structural Determinants of Health Inequities in Maternal Health. *Journal Of Women’S Health (2002)*, *30*(2), 230–235. 10.1089/jwh.2020.8882 PMC8020519 33181043

[CR4] Dehlendorf, C., Harris, L. H., & Weitz, T. A. (2013). Disparities in abortion rates: A public health approach. *American Journal Of Public Health*, *103*(10), 1772–1779. 10.2105/ajph.2013.301339 PMC3780732 23948010

[CR5] Farin, S. M., Hoehn-Velasco, L., & Pesko, M. F. (2021). The impact of legal abortion on maternal health: Looking to the past to inform the present. *Women*.

[CR6] Girardi, G., Longo, M., & Bremer, A. A. (2023). Social determinants of health in pregnant individuals from underrepresented, understudied, and underreported populations in the United States. *International journal for equity in health,**22*(1), 186. 10.1186/s12939-023-01963-x PMC10483713 37674167

[CR7] Gunja, M. Z., Gumas, E. D., & II, R. D. W. (2023). U.S. health care from a global perspective, 2022: Accelerating spending, worsening outcomes. https://www.commonwealthfund.org/publications/issue-briefs/2023/jan/us-health-care-global-perspective-2022

[CR8] Hoyret, D. L. (2024). *Maternal Mortality Rates in the United States, 2022*. NCHS Health E-Stats.

[CR9] Health Resources & Services Administration. (2025) *Healthy start*. U.S. Department of Health and Human Services. https://mchb.hrsa.gov/programs-impact/healthy-start

[CR10] https://www.ncbi.nlm.nih.gov/pmc/articles/PMC2995603/

[CR11] https://www.ncbi.nlm.nih.gov/pmc/articles/PMC2995603/pdf/pcd76A116.pdf

[CR12] Klaiman, T., Chainani, A., & Bekemeier, B. (2016). The importance of partnerships in local health department practice among communities with exceptional maternal and child health outcomes. *Journal of Public Health Management and Practice,**22*(6), 542–549. 10.1097/PHH.0000000000000402 26910874

[CR13] Kroelinger, C. D., Barfield, W. D., & Callaghan, W. M. (2012). Expanding and enhancing federal, state, tribal, and academic partnerships to advance the field of maternal and child health. *Maternal And Child Health Journal*, *16*(Suppl 2), 189–192. 10.1007/s10995-012-1193-6 23196411

[CR14] *Low Birth Weight* (2024). World Health Organization. https://www.who.int/data/nutrition/nlis/info/low-birth-weight

[CR15] Mays, G. P. (2020). S. F. *National Longitudinal Survey of Public Health Systems (NALSYS), [United States], 1998–2018*. 10.3886/ICPSR23420.v4

[CR16] Mays, G. P., & Scutchfield, F. D. (2010). Improving public health system performance through multiorganizational partnerships. *Preventing chronic disease*, *7*(6), A116–A116. https://pubmed.ncbi.nlm.nih.gov/20950523 PMC2995603 20950523

[CR17] Mays, G. P., Mamaril, C. B., & Timsina, L. R. (2016). Preventable Death Rates Fell Where Communities Expanded Population Health Activities Through Multisector Networks. *Health Aff (Millwood)*, *35*(11), 2005–2013. 10.1377/hlthaff.2016.0848 27834240

[CR18] Morgan, I. A., Tucker, C., Urlaub, D. M., Shuler, T. O., & Cilenti, D. (2020). Leveraging an academic-practice partnership to improve maternal and child health outcomes in North Carolina. *North Carolina Medical Journal,**81*(1), 5–13. 10.18043/ncm.81.1.5 31908325

[CR19] Ostovari, M., & Yu, D. (2019). Impact of care provider network characteristics on patient outcomes: Usage of social network analysis and a multi-scale community detection. *PLoS One,**14*(9), Article e0222016. 10.1371/journal.pone.0222016 PMC6733513 31498827

[CR20] Preterm birth. (2023). World Health Organization. https://www.who.int/news-room/fact-sheets/detail/preterm-birth

[CR21] Quinlan, T. A. G., Mitchell, A. L., & Mays, G. P. (2023). Who Delivers Maternal and Child Health Services? The Contributions of Public Health and Other Community Partners. *Milbank Quarterly*, *101*(1), 179–203. 10.1111/1468-0009.12600 PMC10037689 36704906

[CR22] Saleeby, E., Scibetta, E., Greenwell, L., Jackson, A., & Robinson, N. (2021). Mama’s neighborhood: Reimagining prenatal care to support mothers and reduce stress. *NEJM Catalyst*. 10.1056/CAT.20.0654

[CR23] Simpson, K. R. (2023). Effect of the COVID-19 Pandemic on Maternal Health in the United States. *Mcn; American Journal Of Maternal Child Nursing*, *48*(2), 61. 10.1097/nmc.0000000000000895 PMC9951405 36823723

[CR24] Statistics, N. C., & f., H. (2016). *About the national vital statistics system* US centers for disease control and prevention. https://www.cdc.gov/nchs/nvss/about_nvss.htm

[CR25] Trost S. L., Beauregard J., Njie F, (2022). *Pregnancy-related deaths: Data from maternal mortality review committees in 36 US States, 2017–2019.*

[CR26] Ventures, S. (2021). *The U.S. Maternal Vulnerability Index (MVI) Methodology*. The Bill and Melinda Gates Foundation. Retrieved 02/14/2022 from https://mvi.surgoventures.org

[CR27] Vilda, D., Hardeman, R., Dyer, L., Theall, K. P., & Wallace, M. (2021). Structural racism, racial inequities and urban-rural differences in infant mortality in the US. *J Epidemiol Community Health*, *75*(8), 788–793. 10.1136/jech-2020-214260 PMC8273079 33504545

[CR28] Walker, B. (1989). The Future of Public Health: The Institute of Medicine’s 1988 Report. *Journal of Public Health Policy*, *10*(1), 19–31. 10.2307/3342941 2715336

[CR29] Weller, B. E., Bowen, N. K., & Faubert, S. J. (2020). Latent class analysis: A guide to best practice. *Journal of Black Psychology,**46*(4), 287–311. 10.1177/0095798420930932

[CR30] *White House Blueprint addressing the Maternal Health Crisis*. (2022). Retrieved from https://www.whitehouse.gov/wp-content/uploads/2022/06/Maternal-Health-Blueprint.pdf

